# Platelet-Derived Biomaterials Exert Chondroprotective and Chondroregenerative Effects on Diabetes Mellitus-Induced Intervertebral Disc Degeneration

**DOI:** 10.3390/life11101054

**Published:** 2021-10-07

**Authors:** Wen-Cheng Lo, Chun-Chao Chang, Chun-Hao Chan, Abhinay Kumar Singh, Yue-Hua Deng, Chia-Ying Lin, Wen Tsao, Shaw-Ting Chien, Chang-Hsien Lin, Win-Ping Deng

**Affiliations:** 1 School of Medicine, College of Medicine, Taipei Medical University, Taipei 110301, Taiwan; 2 Department of Neurosurgery, Taipei Medical University Hospital, Taipei 110301, Taiwan; 3 Division of Gastroenterology and Hepatology, Department of Internal Medicine, Taipei Medical University Hospital, Taipei 110301, Taiwan; chunchao@tmu.edu.tw; 4 Division of Gastroenterology and Hepatology, Department of Internal Medicine, School of Medicine, Taipei Medical University, Taipei 110301, Taiwan; 5 School of Dentistry, College of Oral Medicine, Taipei Medical University, Taipei 110301, Taiwan; d204107001@tmu.edu.tw (C.-H.C.); abhinaymrdls@gmail.com (A.K.S.); yuehuahua828@gmail.com (Y.-H.D.); 6 Stem Cell Research Center, College of Oral Medicine, Taipei Medical University, Taipei 110301, Taiwan; b228107024@tmu.edu.tw (C.-Y.L.); b228107014@tmu.edu.tw (W.T.); chiens3@uw.edu (S.-T.C.); 7 School of Oral Hygiene, College of Oral Medicine, Taipei Medical University, Taipei 110301, Taiwan; 8 Department of Family Medicine, School of Medicine, College of Medicine, Taipei Medical University, Taipei 110301, Taiwan; 862077@h.tmu.edu.tw; 9 Department of Family Medicine, Taipei Medical University Hospital, Taipei 110301, Taiwan; 10 Graduate Institute of Basic Medicine, Fu Jen Catholic University, New Taipei City 242062, Taiwan; 11 Department of Life Science, Tunghai University, Taichung 407224, Taiwan

**Keywords:** diabetes mellitus (DM), hyperglycemia, intervertebral disc degeneration (IVDD), immortalized human nucleus pulposus cells (ihNPs), platelet-derived biomaterials (PDB), reactive oxygen species (ROS)

## Abstract

Complications of diabetes mellitus (DM) range from acute to chronic conditions, leading to multiorgan disorders such as nephropathy, retinopathy, and neuropathy. However, little is known about the influence of DM on intervertebral disc degeneration (IVDD). Moreover, traditional surgical outcomes in DM patients have been found poor, and to date, no definitive alternative treatment exists for DM-induced IVDD. Recently, among various novel approaches in regenerative medicine, the concentrated platelet-derived biomaterials (PDB), which is comprised of transforming growth factor-β1 (TGF-β1), platelet-derived growth factor (PDGF), etc., have been reported as safe, biocompatible, and efficacious alternatives for various disorders. Therefore, we initially investigated the correlations between DM and IVDD, through establishing in vitro and in vivo DM models, and further evaluated the therapeutic effects of PDB in this comorbid pathology. In vitro model was established by culturing immortalized human nucleus pulposus cells (ihNPs) in high-glucose medium, whereas in vivo DM model was developed by administering streptozotocin, nicotinamide and high-fat diet to the mice. Our results revealed that DM deteriorates both ihNPs and IVD tissues, by elevating reactive oxygen species (ROS)-induced oxidative stress, inhibiting chondrogenic markers and disc height. Contrarily, PDB ameliorated IVDD by restoring cellular growth, chondrogenic markers and disc height, possibly through suppressing ROS levels. These data imply that PDB may serve as a potential chondroprotective and chondroregenerative candidate for DM-induced IVDD.

## 1. Introduction

Intervertebral disc degeneration (IVDD), a common type of musculoskeletal disorder, may lead to spinal instability, neurothlipsis and stenosis, which are the major etiologies of low back pain [1]. To date, the IVDD pathophysiology remains largely unclear and no adequate therapeutic method exists to restore the degenerative intervertebral disc. Diabetes mellitus (DM) is a major public health problem with an estimated incidence of 300 million in 2025 [2]. Around 90% of the cases are type 2 DM (T2DM), which has been revealed with its adverse effects on various organ systems, leading to retinopathy, cardiovascular disease, neuropathy, chronic renal failure, etc. [3]. Particularly, epidemiological reports have observed a higher incidence of IVDD in diabetic patients compared to the healthy ones, the time span of DM has also been found as a risk factor for lumbar disc degeneration [4,5]. Therefore, it is anticipated that a DM-induced hyperglycemic microenvironment may induce an enhanced catabolic metabolism through increased inflammatory response, accumulated cellular senescence, suppressed proliferation, and compromised ability of self-repair.

A few recent reports have indicated that the DM may engender IVDD [6,7], by inducing degenerative changes such as apoptosis, senescence and matrix degradation of nucleus pulposus (NP) cells [8]. These evidences imply that the suppression of high glucose might be a potential strategy for delaying disc degeneration in diabetic patients. However, a paucity of data exists on the influence of hyperglycemia on human IVD cells. Therefore, we initially aimed to examine the impact of DM on IVD-derived NP cells. Further, when compared to non-DM, the surgical outcomes of DM patients undergoing degenerative disc surgery have been found to be poor, due to enhanced incidence of fusion complications and surgical site infection [9,10,11]. To avoid these adverse events, in recent years, biological approaches including bioactive factors have been developed and have shown promising results [12], and therefore may be an ideal therapeutic option for degenerative IVD.

Recently, being autologous, platelet-derived biomaterials (PDB) have been reported as a safe, biocompatible and efficacious alternative for improving diabetes-inducing cardiac pathophysiological changes [13] and diabetic foot ulcers [14]. Based on these indications, in this study, we applied PDB therapy in DM-induced IVDD. Blood-derived platelet-rich plasma (PRP) contains several concentrated growth factors including platelet-derived growth factor (PDGF), vascular endothelial growth factor (VEGF), insulin-like growth factor-1 (IGF-1), transforming growth factor-β1 (TGF-β1), platelet-derived angiogenesis factor (PDAF) and epidermal growth factor (EGF), which are released upon the activation or physical disruption of α-granule of platelet [15]. This releasate is designated as PDB, and has been indicated to stimulate chondrocyte proliferation and proteoglycan biosynthesis, which was mostly attributed to TGF-β1 and PDGF [16]. Also, PDB could significantly inhibit the levels of tumor necrosis factor (TNF)-α and interleukin (IL)-1β in chondrocytes and NP cells, indicating its anti-inflammatory effects [17,18]. Additionally, we have previously demonstrated the ameliorative effects of PDB on nicotine-induced IVDD [19], which revealed its potential as a cure for chronic-inflammation-induced IVDD.

Based on the abovementioned evidence, we hypothesized whether PDB could retard degenerative IVD characteristics under a hyperglycemic microenvironment. During in vitro studies, we established the DM model by treating immortalized human nucleus pulposus cells (ihNPs) with high-glucose medium (HGM), which was later treated by PDBs. The therapeutic effect of PDB was also evaluated in the in vivo DM-IVDD mice model, which was created through the treatment of streptozotocin (STZ) + nicotinamide (NA) and a high-fat diet. The effects of PDB in DM-IVDD were investigated in terms of its anti-inflammatory, anti-oxidative, and chondroprotective potential, by monitoring specific biomarkers at gene and protein levels, as well as histologic assessments.

## 2. Materials and Methods

### 2.1. Cell Culture

In this study, the immortalized human nucleus pulposus cells (ihNPs) expressing normal chondrocyte-like phenotypes were used as the cell model [20]. Briefly, human NPs were harvested from healthy IVD of a 39-year-old male donor, and the isolated human NPs were then immortalized through transfecting with retroviral vector HPV16 E6/E7 [21,22]. For routine cultures, ihNPs were maintained in DMEM/F12 medium and passaged at a 1:3 ratio when the cells appeared subconfluent.

### 2.2. Cell Viability and Proliferation

Cell viability was evaluated through MTT assay, in which 1 × 10^3^ cells/well of ihNPs were seeded into 96-well plate, then incubated in high-glucose medium (HGM), with or without PDB treatment (TGF-β1 = 0.1 or 2 ng/mL), for 1, 3, 5 and 7 days; the medium was replaced every two days. Thereafter, the medium was discarded and replaced with 100 μL of MTT solution (Sigma, Saint Louis, MO, USA), then incubated for 4 h at 37 °C. Next, MTT solution was replaced with DMSO to dissolve the dark blue formazan crystal, and the absorbance was measured at 595 nm using ELISA reader (Multiskan RC Microplate Reader, Thermo). For evaluating cell proliferation, 5 × 10^5^ cells/well of ihNPs were seeded into six-well plates, then incubated in control (DMEM/F12 + 10% FBS), glucose, or glucose + PDB medium according to their experimental groups. After 5 days of treatment, cell numbers of each group were determined through the automated cell counter Countess™ (Life Technologies, Carlsbad, USA).

### 2.3. Platelet-Derived Biomaterials (PDB) Preparations

The isolation and quantification of PDB have been described in our previous study [23]. In brief, the whole blood was harvested from the Taipei Blood Center and separated using the MCS blood cell separation system (Haemonetics Corp., Braintree, MA, USA). Next, bovine thrombin was employed then centrifuged with 3000 rpm for 6 min at room temperature to remove aggregated fibrin, PDB was then collected and stored at −20 °C. The concentrations of PDB were further quantified by the expression of TGF-β1 through enzyme-linked immunosorbent assay (ELISA) (#DB100, R&D systems, Minneapolis, MN, USA).

### 2.4. Real-Time Polymerase Chain Reaction (qPCR)

After harvesting cells from each experimental group, the total RNA was isolated by a High Pure RNA Isolation Kit (Roche, Germany), following the manufacturer’s protocol. Reverse transcription-PCR (RT-PCR) was conducted as previously described [24]. Later, qPCR assay was performed using the ABI 7300 real-time detection PCR system (Applied Biosystems, USA), and the relative gene expressions were obtained through the 2^−ΔCt^ or 2^−ΔΔCt^ method with calibration samples in each experiment. The utilized primers for each gene are listed as following:

SOX9— Forward: AGACCTTTGGGCTGCCTTAT;
  Reverse: TAGCCTCCCTCACTCCAAGA
Col II— Forward: CCTTCCTGCGCCTGCTGTC;
  Reverse: GGCCCGGATCTCCACGTC
Aggrecan— Forward: CCGCTACGACGCCATCTG;
  Reverse: CCCCCACTCCAAAGAAGTTTT
β-actin— Forward: AGAGCTACGAGCTGCCTGAC;
  Reverse: AGCACTGTGTTGGCGTACAG

### 2.5. Western Blotting Analysis

For isolating protein from ihNPs and IVD tissue, the ihNPs were harvested and the spinal discs were directly excised from mice. Both ihNPs and disc tissue were then mixed with radioimmunoprecipitation assay (RIPA) buffer (RIPA lysis buffer, Millipore 20–188) and incubated on ice for 30 min, then further sonicated by ultrasonic liquid homogenizer (Misonix XL-2000). Thereafter, the cell suspension was centrifuged at 13,000 g for 20 min at 4 °C, the supernatant was collected as cell lysate. Protein content was determined through protein assay dye (BioRad, #500-0006) and mixed with 6× protein sample dye as loading samples. Afterwards, the prepared samples were denatured for 10 min at 100 °C, then gel electrophoresis was performed for separating target proteins. The separated proteins were then transferred to polyvinylidene difluoride (PVDF) membrane (IPVH00010, Millipore, Bedford, MA, USA), and later blocking was conducted with 4% bovine serum albumin (BSA) blocking buffer for 1 h at room temperature. The membranes were further incubated with primary antibodies including Sox9 (ab59252, 1:500, Abcam, Cambridge, UK), type 2 collagen (Col II) (ab34712, 1:1000, Abcam), AGN (MABT83, 1:500, Millipore, Billerica, MA, USA) and β-actin (GTX109639, 1:10000, GeneTex) antibodies. Later, secondary antibodies including anti-rabbit IgG (H + L) antibody, peroxidase-labeled (04-15-06, 1:5000, Kirkegaard & Perry Laboratories, USA) and anti-mouse IgG (H + L) antibody, human serum adsorbed and peroxidase-labeled (04-18-06, 1:5000, Kirkegaard & Perry Laboratories, USA) were applied and incubated for 1 h at room temperature. The protein bands were then visualized through a chemiluminescence detection kit (WBKLS0500, Millipore, Billerica, MA, USA), and images were captured by Mutigel-21 (Fluorescent Gel Image System, TopBio, Taipei, Taiwan).

### 2.6. Determination of Reactive Oxygen Species (ROS)

For detecting ROS level (oxidative stress marker), the ihNPs were collected and centrifuged at 10,000 g for 5 min. The supernatant was then collected for quantifying the concentration of ROS using an OxiSelect™ In Vitro ROS/RNS Assay Kit (SAT-347, Cell Biolabs, USA) in accordance with the manufacturer’s protocol. Briefly, the level of generated intracellular ROS was detected using 2′, 7′-dichlorofluorescein diacetate (DCFH-DA). Next, 50 μL of sample and catalyst were added into each well of a 96-well plate, mixed well and incubated for 5 min at room temperature. Afterward, 100 μL of DCFH solution were added and incubated for 15–45 min at room temperature and prevented from light exposure. Lastly, the fluorescence was measured with a Microplate Reader SkanIt (Thermo) plate reader at 480 nm excitation/530 nm emission.

### 2.7. Animal Study

All animal studies and care procedures were followed with the guidelines approved by the Institutional Animal Care and Use Committee (IACUC) of Taipei Medical University. Four-week-old male C57BL/6 mice were purchased from the National Laboratory Animal Center, Taipei, Taiwan. All the mice were maintained in pathogen-free conditions at 25 °C, humidity level (64–68%), and fed with autoclaved food and water. After one week of acclimatization, the mice were divided into 3 groups: non-DM control (n = 5), DM group (n = 5), and DM + PDB group (n = 5). For establishing DM, the mice were intraperitoneally injected with 55 mg/kg of streptozotocin (STZ) and 100 mg/kg of nicotinamide (NA) on day 1 and day 3, then fed with a high-fat diet, while the non-DM control group was injected with PBS buffer. The body weight and fasting blood glucose (FBG) levels were measured every week. After FBG reached diabetic level, an oral glucose tolerance test (OGTT) and intraperitoneal insulin tolerance test (IPITT) were performed for confirming the successful establishment of DM. After the mice fasted for 8–12 h, 1 g/kg of glucose was orally administered for OGTT, and 0.75 IU/kg body weight of insulin (Humulin^®^ R Regular insulin human injection) was given intraperitoneally for IPITT. Next, the blood was collected from the tail at 0, 30, 60 and 90 min for both OGTT and IPITT. Blood glucose levels were then determined using a glucometer (Easy TouchGU, Taiwan). After 8 weeks of DM induction, 100 µL of PDB (2 ng/mL) were subcutaneously administered to the skin above the lumbar spine (n = 5) once per week for 4 weeks. After PDB treatment, mice were euthanized and the spines of each group were then isolated for histological and immunohistological studies.

### 2.8. Histological and Immunohistological Analysis

The hematoxylin and eosin (H&E) staining of tissue sections was conducted using the H&E Staining Kit (Abcam, ab245880), according to the manufacturer’s protocol. Immunohistochemistry (IHC) staining was performed through the avidin-biotin peroxidase detection method. Briefly, the tissue sections were deparaffinized with xylene and rehydrated using decreasing concentrations of ethanol, and were later blocked by 4% bovine serum albumin (BSA) to avoid non-specific binding. Further, a commercial avidin-biotin blocking kit (Vector Laboratories, USA) was utilized to block the avidin and biotin binding sites. Thereafter, sections were incubated with their respective antibodies for 30 min at room temperature, followed by overnight incubation at 4 °C. Later, the sections were then rinsed with ice-cold saline and further incubated with secondary biotinylated anti-mouse immunoglobulin G. Afterwards, 0.3% of H_2_O_2_ in methanol was used to block the endogenous peroxidase activity, and horseradish peroxidase activity was visualized by diaminobenzidine (Vector Laboratories) reaction. Additionally, sections were also stained with alcian blue to determine the levels of glycosaminoglycans (GAG). The stainings were then quantified by Image J (NIH, https://imagej.nih.gov/ij/download.html, accessed on 18 June 2020). Disc height index (DHI) was assessed using the method as described previously [25]. Briefly, DHI was calculated by the following formula: DHI = AC + BD/CE + DF (as shown in Figure 5D). The changes of DHI were calculated and normalized using the following formula: % DHI = (postoperative DHI/preoperative DHI) × 100%. The DHI illustration was depicted from the free online resource Wikipedia (https://commons.wikimedia.org/wiki/File:716_Intervertebral_Disk.svg, accessed on 13 December 2020), with further modification for describing DHI calculation.

### 2.9. Statistical Analysis

Data are presented as mean ± SD. The experiments were performed in at least three biological replicates if no further stated. The statistical analysis of data was done with Student’s *t-test*, one-way and two-way ANOVA (GraphPad Prism 8). *p*-value <0.05 was considered statistically significant. The symbols *, **, and *** represent *p* < 0.05, *p* < 0.01, and *p* < 0.001, respectively.

## 3. Results

**Figure 1:**
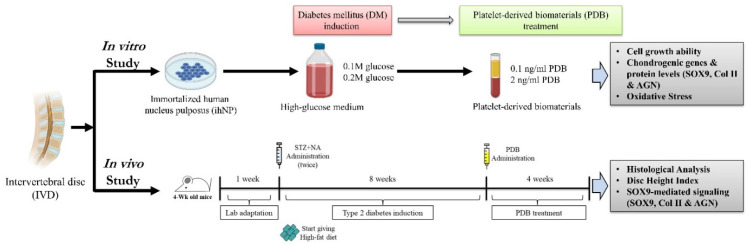
Schematic experimental design of PDB therapy in DM-induced IVDD in vitro and in vivo. PDB: Platelet-derived biomaterials, DM: Diabetes mellitus, IVDD: Intervertebral disc degeneration. Illustration created with BioRender.com, accessed on 25 September 2020.

To investigate the repair and regenerative effects of platelet-derived biomaterials (PDB) on DM-induced IVDD, the in vitro and in vivo DM models were established and further treated with PDB, the experimental schematic of which has been illustrated in Figure 1.

### 3.1. High Glucose Suppresses Chondrogenic Potential of ihNPs

**Figure 2:**
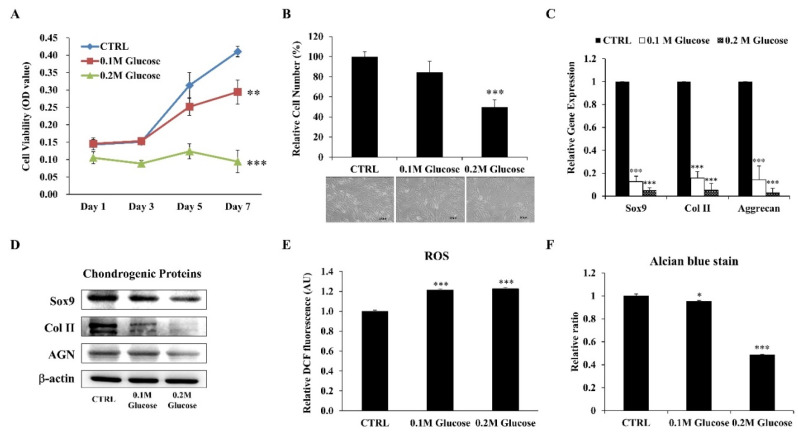
Effects of HGM on ihNPs cell growth ability, oxidative stress and chondrogenic markers. After treating with HGM (0.1 M and 0.2 M glucose), the (**A**) cell viability and (**B**) cell number along with cell morphology of ihNPs were evaluated. Chondrogenic markers including SOX9, Col II and AGN were also determined at both (**C**) gene and (**D**) protein levels. The relative expressions of (**E**) ROS and (**F**) alcian blue staining were further determined for detecting cellular oxidative stress and GAGs, respectively. Results are presented as mean ± S.D. (n = 3; * *p* < 0.05, ** *p* < 0.01, *** *p* < 0.001). HGM: High-glucose medium, Col II: Type 2 collagen, AGN: Aggrecan, ROS: Reactive oxygen species, GAGs: Glycosaminoglycans.

The in vitro DM model was established by culturing ihNPs in high-glucose medium (HGM) with different glucose concentrations (0.1 M and 0.2 M) to mimic the diabetic condition. The effects of HGM on ihNPs were later evaluated by growth ability, chondrogenic markers, oxidative status and alcian blue staining. Our results displayed an inhibited cell viability (Figure 2A) and cell number (Figure 2B) of ihNPs, particularly in the 0.2 M glucose group. Additionally, gene (Figure 2C) and protein (Figure 2D) levels of chondrogenic markers including SOX9, type 2 collagen (Col II) and aggrecan (AGN) were significantly decreased, indicating the chondro-inhibitory nature of hyperglycemia. Reactive oxygen species (ROS), which is well known to induce cellular oxidative stress, chondrocyte hypertrophy and apoptosis [26], was also significantly increased in HGM groups (Figure 2E). Lastly, alcian blue staining indicating the presence of glycosaminoglycans in chondrocyte was highly decreased in HGM groups, particularly in the 0.2 M glucose group (Figure 2F). Collectively, these data showed significantly inhibited chondrogenic potential of ihNPs in the dose of 0.2 M glucose, and hence we chose this dose for the subsequent experiments.

### 3.2. Chondroprotective Effects of Platelet-Derived Biomaterials (PDB) in the HGM-Treated ihNPs

**Figure 3:**
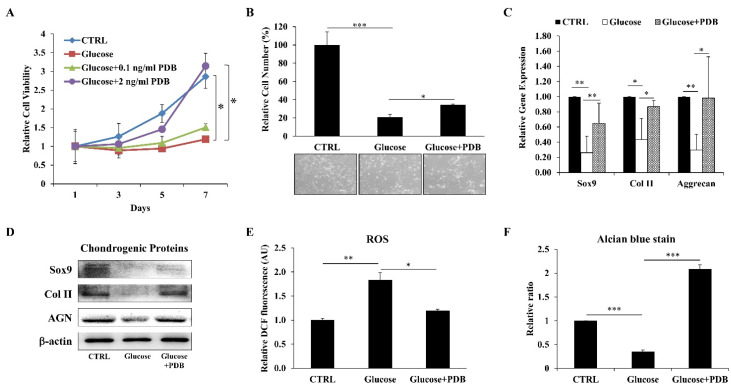
Impact of PDB on cell growth, oxidative stress and chondrogenesis in HGM-treated ihNPs. ihNPs were treated with PDB in the presence of HGM (0.2 M), and (**A**) cell viability, the (**B**) cell number, (**C**) gene and (**D**) protein levels of chondrogenic markers (SOX9, Col II and AGN) were determined. Also, the expressions of (**E**) ROS and (**F**) alcian blue staining were further examined. Results are presented as mean ± S.D. (n = 3; * *p* < 0.05, ** *p* < 0.01, *** *p* < 0.001). PDB: Platelet-derived biomaterials, HGM: High-glucose medium, Col II: Type 2 collagen, AGN: Aggrecan, ROS: Reactive oxygen species.

After confirming degenerative characteristics of high glucose on ihNPs, we supplemented PDB into HGM for evaluating its protective effect under a hyperglycemic microenvironment. Our results revealed a significantly recovered cell viability (Figure 3A) and cell number (Figure 3B) of HGM-treated ihNPs after one week of PDB (2 ng/mL) treatment. Also, PDB remarkably increased the expression of chondrogenic markers (SOX9, Col II and Aggrecan) at gene (Figure 3C) and protein (Figure 3D) levels. Moreover, ROS (oxidative stress biomarker) was also suppressed (Figure 3E). These chondroprotective effects of PDB were further validated through alcian blue staining results (Figure 3F), indicating increased accumulation of glycosaminoglycans.

### 3.3. Establishment of Streptozotocin + Nicotinamide (STZ + NA) and High-Fat Diet-Induced Type 2 Diabetes Mellitus (T2DM) Mice Model

**Figure 4:**
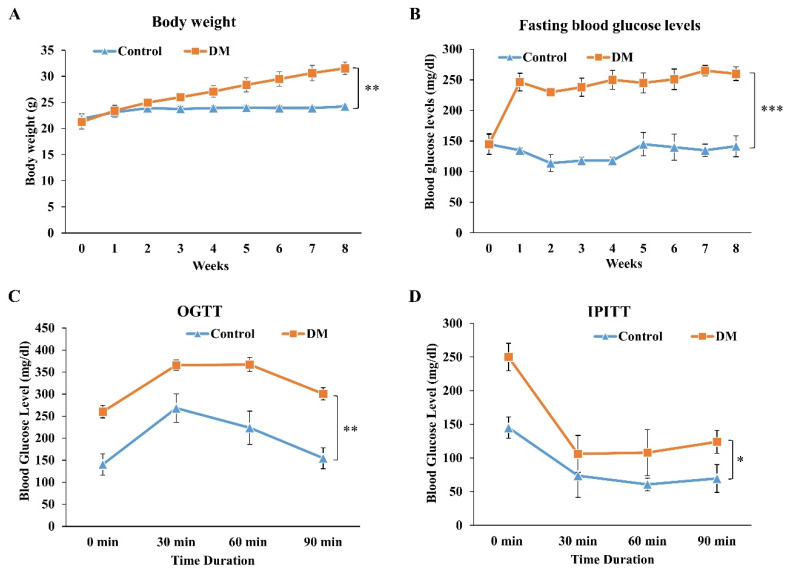
Establishment and validation of STZ + NA and high-fat diet-induced T2DM mice model. To validate the successful establishment of T2DM mice model, the (**A**) body weight, (**B**) fasting blood glucose levels, (**C**) OGTT and (**D**) IPITT were evaluated in STZ + NA and high-fat diet-induced T2DM mice. Results are presented as mean ± S.D. (n = 3; * *p* < 0.05, ** *p* < 0.01, *** *p* < 0.001). STZ: Streptozotocin; NA: Nicotinamide, T2DM: Type 2 diabetes mellitus, OGTT: Oral glucose tolerance test, IPITT: Intraperitoneal insulin tolerance test.

To investigate the in vivo therapeutic effect of PDB on IVD degeneration, we firstly established T2DM C57BL/6 mice by administering STZ + NA and high-fat diet, as previously described [27]. During the T2DM induction period (as shown in Figure 1), the body weight and fasting blood glucose levels of mice were measured on a weekly basis. Diabetic mice showed an increasing trend of body weight (Figure 4A), along with their fasting blood glucose levels, which were higher than 200 mg/dL since the first week (Figure 4B). The diabetic profile of mice was further confirmed through an oral glucose tolerance test (OGTT) and intraperitoneal insulin tolerance test (IPITT), showing poor clearance of circulating glucose (Figure 4C) and insulin tolerance (Figure 4D) of DM mice. These results indicated the successful establishment of a stable T2DM mice model.

### 3.4. PDB Ameliorates T2DM-Induced IVDD In Vivo

**Figure 5:**
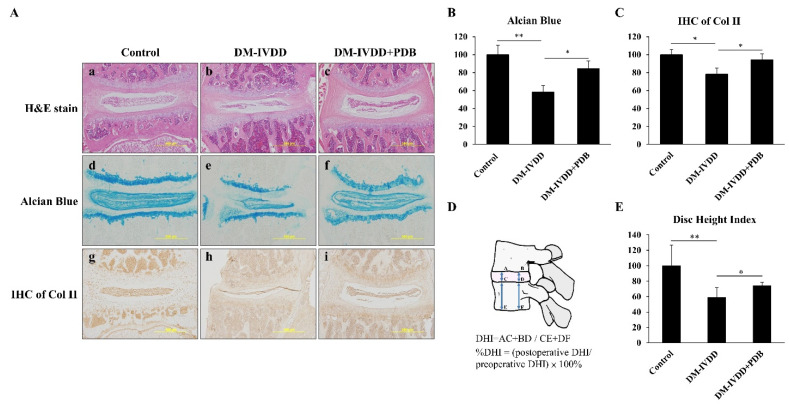
PDB therapy in DM-induced IVDD mice model. After 8 weeks of DM induction, PDB (2 ng/mL) was subcutaneously administered to the skin above the lumbar spine (n = 5) once per week for 4 weeks. After PDB treatment, the spines were harvested for histological and immunohistological studies. (**A**) Representative hematoxylin & eosin (H&E) staining (**a**–**c**, upper panel), alcian blue staining (**d**–**f**, middle panel), and immunohistochemical staining of Col II (IHC of Col II) (**g**–**i**, lower panel) in the control, DM-IVDD and DM-IVDD + PDB groups. The expressions of (**B**) alcian blue and (**C**) IHC of Col II were further quantified through Image J. (**D**) The illustration and calculation formula of DHI values, and their relative fold changes, were further quantified (**E**). The results are presented as mean ± S.D. (n = 5; *p < 0.05, ** *p* < 0.01, *** *p* < 0.001). PDB: PDB: Platelet-derived biomaterials, DM: Diabetes mellitus, DM-IVDD: Diabetes mellitus-induced intervertebral disc degeneration, DHI: Disc height index.

To investigate the effects of T2DM and PDB on IVDD, PDB (2 ng/mL) was subcutaneously injected once per week for 4 weeks, to the skin above the lumbar spine (n = 5), due to greater geometrical [28] and torsional [29] resemblance of lumbar segments of mice to the human disc. Later, the spines of each mouse were isolated and sectioned for H&E, alcian blue, and IHC staining of Col II. Our H&E staining results revealed that, compared to the control (Figure 5A(a)), a narrowed intervertebral space and impaired morphology of nucleus pulposus in DM group (Figure 5A(b)) were observed, but were improved in the PDB-treated group (Figure 5A(c)). Alcian blue-stained glycosaminoglycans were also higher in the control group (Figure 5A(d)), which were suppressed in the DM group (Figure 5A(e)). However, glycosaminoglycans contents were improved in the PDB-treated group (Figure 5A(f)). Similar to the above results, the IHC staining signals of type II collagen (Col II) were found normal in the control group (Figure 5A(g)), highly diminished in the DM group (Figure 5A(h)), but recovered in the PDB-treated group (Figure 5A(i)). These results were further substantiated through the quantitative results (Figure 5B, C). The normal IVD can be measured in the terms of disc height index (DHI) [30], as shown by the specific formula in Figure 5D. Our results reveal that DHI was significantly reduced in the DM group, but elevated in PDB treatment (Figure 5E), which is also in agreement with staining results.

### 3.5. PDB Restores Chondrogenic Markers in T2DM-Induced IVDD Mice Model

**Figure 6:**
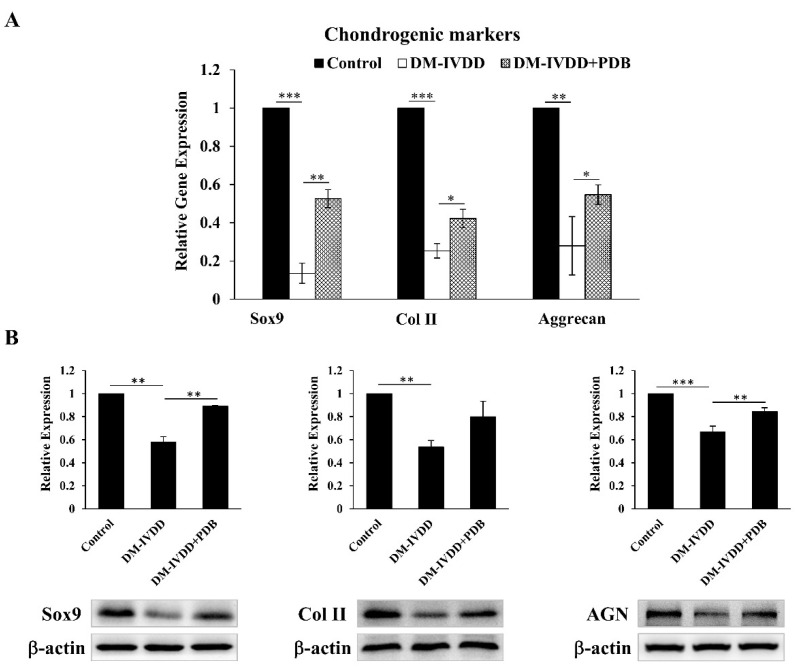
Chondrogenic regulation of PDB therapy in DM-induced IVDD mice. (**A**) Gene and (**B**) protein expressions of chondrogenic markers (SOX9, Col II and AGN) in control, DM-IVDD and DM-IVDD + PDB groups. Results are presented as mean ± S.D. (n = 3; * *p* < 0.05, ** *p* < 0.01, *** *p* < 0.001). DM: Diabetes mellitus, DM-IVDD: Diabetes mellitus-induced intervertebral disc degeneration.

To further substantiate the regenerative effects of PDB, the levels of chondrogenic factors including SOX9, type II collagen and aggrecan were determined in disc tissues. Our results demonstrated that, compared to the control, gene expressions of SOX9, Col II and AGN were significantly inhibited in the DM-IVDD group, which were recovered in the DM-IVDD + PDB group (Figure 6A). In line with these results, the protein expression of SOX9, Col II and AGN also shared a similar trend (Figure 6B). These results revealed the restorative effects of PDB on chondrogenic markers in the DM-IVDD model.

## 4. Discussion

This is the first report on PDB therapy in DM-induced IVDD, in which we initially characterized the hyperglycemia-induced catabolic characteristics in the IVD-derived ihNPs cultured under high-glucose conditions, simulating a diabetic state. During in vivo investigations, STZ + NA and HFD-induced DM mice showed structural malformation of IVD, in the terms of narrowed disc height and highly reduced levels of chondrogenic markers including SOX9, type 2 collagen and aggrecan at gene and protein levels, representing IVDD. These results are in line with a prospective study of 24 patients, T2DM hastened stress-induced deformed NP with clustered chondrocytes and enhanced cellularity, leading to IVD senescence and disc degeneration [31]. A retrospective study also concluded that prolonged T2DM and its bad control could result in severe disc degeneration [5]. Kakadiya et al. also found that DM patients exhibited worse IVD degeneration than non-DM patients, showing increased disc apoptosis and matrix aggrecan fragmentation [32]. Interestingly, a statistically lower IVD height between the 2nd and 3rd lumbar vertebrae has been reported in diabetic patients [33]. In a similar trend, high glucose-induced excessive ROS could promote apoptosis of cartilage endplate cells, which are also considered as one of the initiators of IVDD [7]. Other studies have evidenced that the levels of chondrogenic transcription factor SOX9 and ECM proteins including collagen type II, aggrecan and glycosaminoglycans (GAGs) are suppressed under hyperglycemic conditions [34,35,36]. Collectively, DM may degenerate IVD possibly through inducing excessive ROS and downregulating SOX9-mediated chondrogenic signaling pathway, eventually leading to the degeneration of IVD.

In our study, however, PDB treatment revealed ameliorative effects on DM-induced pathogenic effects in ihNPs as well as in the mice by elevating chondrogenesis and restored IVD ultrastructure, signifying its therapeutic potential on degenerative IVD. Since the IVDD is induced through diabetic state, PDB may improve pathologic characteristics of IVDD via inhibiting increased blood glucose, malondialdehyde levels (oxidative marker), and elevating the magnitude of islet insulin secretion and antioxidant enzyme [37]. Generally, oral treatments combining anti-inflammatory and anti-AGE drugs could repress advanced glycation end product (AGE)-induced ROS and inflammation in the slowly progressive degenerative spine in diabetes [36]. In agreement with the abovementioned studies, we have also shown that PDB could inhibit hyperglycemia-induced ROS levels, which may lead to reduced activity of matrix-degrading enzymes [38,39,40]. Reportedly, PRP may impart chondroprotective and chondro-regenerative activities through elevating autophagy, anti-inflammatory markers, and suppressing cellular apoptosis in human osteoarthritic cartilage [41]. PRP has also been shown to exert positive effects on DM-mediated reproductive complications. Specifically, PRP could also inhibit testicular damage by significantly increasing spermatogenic cells and the thickness of seminiferous tubules [42]. PRP counteracts the detrimental effect of high-glucose concentrations on mesenchymal stem cells from Bichat fat pad [43]. In accordance with these studies, PDB seems to restore suppressed collagen and proteoglycan contents in the DM-IVDD group by stimulating angiogenesis, cell proliferation and differentiation, whereas reducing pro-apoptotic Bcl-2 gene expression and inhibiting apoptosis [44,45].

Besides, though intradiscal injection for treating IVDD has been commonly investigated, there are certain risks due to its complicated structure compared with other musculoskeletal tissues such as tendons, ligaments, or joints. In an important report, notable risks of intradiscal injection of platelet-rich plasma for the treatment of spondylodiscitis include the risk of transferring dormant bacteria into the discs during administration [46]. To our knowledge, the studies of subcutaneously injected therapy in mice model of IVDD are still very limited and need further investigation. However, previous studies have revealed that subcutaneously injected platelet-rich plasma could exhibit not only the antidiabetic activity and enhanced healing of diabetic oral mucosal wounds [47], but also promote cartilage viability through increasing vascularity of periphery vessels to obtain nutrients and anabolic agents [48]. Since hyperglycemia and hypercholesterolemia of DM may lead to calcification of blood vessels [49,50], our PDB therapy may exert antidiabetic, hypercholesterolemic, as well as anti-IVDD activity.

Apart from various therapeutic outcomes, our study includes some limitations. Though we could not determine in vivo ROS levels and their association with SOX9, previous research has revealed the inhibition of SOX9 by hydrogen peroxide-induced ROS in articular chondrocytes [51]. Similarly, a reduced expression of SOX9 and type II collagen by tert-butylhydroperoxide (oxidative stress inducer) has also been reported, which might occur through the MEK/ERK signaling pathway [52]. These studies support our results showing elevated ROS and inhibited SOX9, type 2 collagen, and aggrecan levels under hyperglycemic conditions. Further, the detailed SOX9-mediated signaling pathway was not fully investigated; however, previous research has revealed the prominent role of SOX9 in regulating IVD degeneration [53]. Next, though we did not measure the blood glucose levels after the PDB therapy, previous studies have indicated the anti-hyperglycemic effects of platelet-contained growth factors [37,54], which may contribute to its therapeutic effect in DM-induced IVDD.

**Figure 7:**
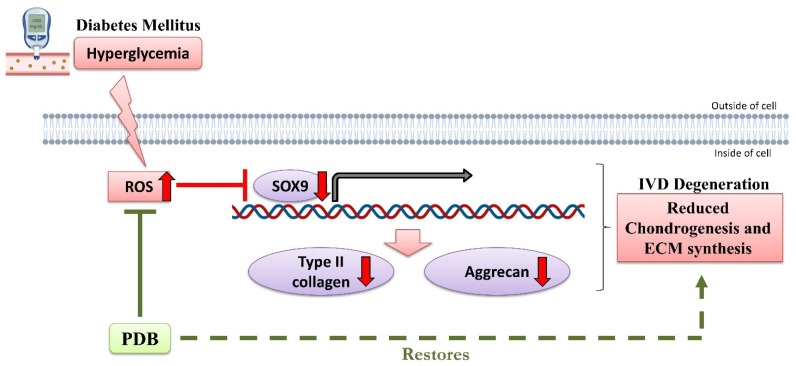
Possible signaling pathway of therapeutic PDB in DM-induced IVDD. Up and down red-colored arrows indicate upregulated and downregulated levels during DM. PDB: Platelet-derived biomaterials, DM-IVDD: Diabetes mellitus-induced intervertebral disc degeneration, ROS: Reactive oxygen species. The figure has been created with BioRender.com, accessed on 11 January 2021.

Conclusively, our results demonstrated that DM may induce IVDD possibly through oxidative stress-mediated inhibition of ECM synthesis and chondrogenesis, as revealed by reduced chondrogenic transcription factor SOX9 and ECM proteins including collagen type II and aggrecan. However, PDB treatment rescued the DM-induced IVD degeneration by suppressing ROS accumulation and restoring chondrogenesis markers (Figure 7).

## Data Availability

Not applicable.
